# Identification of ACKR4 as an immune checkpoint in pulmonary arterial hypertension

**DOI:** 10.3389/fimmu.2023.1153573

**Published:** 2023-06-28

**Authors:** Chen-Yu Jiang, Li-Wei Wu, Yi-Wei Liu, Bei Feng, Lin-Cai Ye, Xu Huang, Yang-Yang He, Yi Shen, Yi-Fan Zhu, Xing-Liang Zhou, Dai-Ji Jiang, Hai-Kun Qi, Hao Zhang, Yi Yan

**Affiliations:** ^1^ Shanghai Clinical Research Center for Rare Pediatric Diseases, Shanghai Children’s Medical Center (SCMC), School of Medicine, Shanghai Jiao Tong University, Shanghai, China; ^2^ Heart Center and Shanghai Institute of Pediatric Congenital Heart Disease, Shanghai Children’s Medical Center (SCMC), School of Medicine, Shanghai Jiao Tong University, Shanghai, China; ^3^ School of Pharmacy, Henan University, Kaifeng, Henan, China; ^4^ School of Biomedical Engineering, Shanghaitech University, Shanghai, China

**Keywords:** pulmonary arterial hypertension, atypical chemokine receptor 4, transcriptomics, chemokines, immune

## Abstract

**Objective:**

Inflammation is recognized as a contributor in the development of pulmonary arterial hypertension (PAH), and the recruitment and functional capacity of immune cells are well-orchestrated by chemokines and their receptors. This study is aimed at identification of critical chemokines in the progression of PAH *via* transcriptomic analysis.

**Methods:**

Differentially expressed genes (DEGs) from lungs of PAH patients were achieved compared to controls based on Gene Expression Omnibus (GEO) database. Gene set enrichment analysis (GSEA) was applied for functional annotation and pathway enrichement. The abundance of immune cells was estimated by the xCell algorithm. Weighted correlation network analysis (WGCNA) was used to construct a gene expression network, based on which a diagnostic model was generated to determine its accuracy to distinguish PAH from control subjects. Target genes were then validated in lung of hypoxia-induce pulmonary hypertension (PH) mouse model.

**Results:**

ACKR4 (atypical chemokine receptor 4) was downregulated in PAH lung tissues in multiple datasets. PAH relevant biological functions and pathways were enriched in patients with low-ACKR4 level according to GSEA enrichment analysis. Immuno-infiltration analysis revealed a negative correlation of activated dendritic cells, Th1 and macrophage infiltration with ACKR4 expression. Three gene modules were associated with PAH *via* WGCNA analysis, and a model for PAH diagnosis was generated using CXCL12, COL18A1 and TSHZ2, all of which correlated with ACKR4. The ACKR4 expression was also downregulated in lung tissues of our experimental PH mice compared to that of controls.

**Conclusions:**

The reduction of ACKR4 in lung tissues of human PAH based on transcriptomic data is consistent with the alteration observed in our rodent PH. The correlation with immune cell infiltration and functional annotation indicated that ACKR4 might serve as a protective immune checkpoint for PAH.

## Introduction

Pulmonary arterial hypertension (PAH), characterized with pulmonary vessel proliferation and extracellular matrix remodeling (1–4), is still an incurable disease with various etiology and pathogenesis (1, 5). In contrast to previous studies directly focusing on the vascular cells and extracellular matrix, growing attention has been shifted to inflammation and other related issues in recent years. Inflammation, including macrophages recruited early in PAH and activities of various cytokines or chemokines, is being deemed as a major contributor to the pathogenesis of PAH (3). Several researchers have indicated that a wide variety of chemokine systems comprising the CCL2-CCR2, CX3CL1-CX3CR1 and CCL5-CCR5 axis involved in macrophage recruitment and pulmonary vascular remodeling (6–8). Even several chemokines, for example, CCL2 and CXCL10, have emerged as potential biomarkers of PAH due to the elevating trend in disease setting and their correlation with disease severity in different forms of PAH (9, 10).

As mentioned above, most of chemokines with increasing circulating levels are deemed as an accelerator because of participation in inflammation amplification during PAH progression (3). However, several chemokines and those receptors may play a protective role in elimination of inflammation. For example, atypical chemokine receptor 4 (ACKR4), familiar with name of CCR11 or PPR1, was originally isolated from bovine papillary tissue in gustatory receptors study (11). However, with further research, several studies have presented a phenomenon of higher ACKR4 expression in lung than in tongue (12). ACKR4, as a chemokine scavenger receptor, mediates the rapid internalization and degradation of chemokines, which is involved in the regulation of CCR7-dependent dendritic cell migration and elimination of homeostatic chemokines to limit Th17 cell-mediated overreaction, especially in tumor environment (13). Nevertheless, it is worth mentioning that the biological effect of ACKR4 in PAH progression is largely unknown.

In this study, we retrieve the gene profiling of lung samples from pulmonary hypertension (PH) patients and corresponding controls from gene expression omnibus (GEO). It was found that the gene expression of ACKR4 was lower in PAH patients than controls. A significantly lower expression of ACKR4 was displayed in PH mice and we speculated that the downregulation of ACKR4 may facilitate the progression of PAH, which might be an effective target for PAH prevention, surveillance and therapy.

## Materials and methods

### Acquisition of transcriptomic data

The transcriptomic data of human pulmonary hypertension (PH) lung samples were obtained from GEO database. In GSE113439, a total of 26 lung samples were obtained, including 15 PH samples (6 idiopathic PAH samples, 4 PAH secondary to connective tissue disease samples, 4 PAH secondary to congenital heart disease samples and 1 chronic thromboembolic pulmonary hypertension sample) and 11 controls (14). In GSE53408, a total of 23 lung samples were obtained to perform the following analyst, composing of 12 PAH and 11 controls (15). In GSE117261, a total of 83 lung samples were extracted to perform analysis process, including 58 PAH and 25 controls (16). Gene profiling of lung samples from 62 PH patients and 22 controls was contained in GSE24988. All gene expression microarray data were downloaded from GEO database in a form of gene expression matrix, which was standardized and quality controlled.

### Identification of differentially expressed genes

We used the limma R package to perform the differential gene analysis as previously described (17, 18). Chemokine related genes were listed in **Table S1**. The following selection criteria were used to screen out differentially expressed genes (DEGs): ∣log FC∣> 0.25 between two groups and adjusted *P* value < 0.05. The *t* test method was used to calculate *P* value of genes, and the adjusted *P* value was calculated by Benjamini and Hochberg’s method. The results of differential gene analysis were visualized by volcano plot and heatmap conducted by ggplot2 R package (19).

### Enrichment analysis

Gene ontology (GO), including three modules of biological processes, molecular functions and cellular components, can be used to annotate and analyze the uploaded gene list, so as to explore the functions involved in genes (20). The clusterProfiler R package were used to perform analysis, and the results of analysis were visualized by ggplot2 R package (21). The false discovery rate (FDR) of results < 0.05 was considered to be statistically significant.

Gene Set Enrichment Analysis (GSEA) is a method for enrichment and analysis of gene sets with different biological states or phenotypes, which can be used to obtain functional differences or pathway differences between gene sets of different biological states or phenotypes (22). The GO database was used to explore functional differences, and Kyoto Encyclopedia of Genes and Genomes (KEGG) or Reactome database was used to explore pathway differences (23). The clusterProfiler R package was used to perform GSEA, and nom *P* value < 0.05 was statistically significant.

### Immune infiltration analysis

Immune infiltration analysis is to weigh the immune related genes in the gene expression matrix to get the activity status of related immune cells and pathways, which can be used to demonstrate relation of interested genes and immune status. The xCell R package was used to perform analysis, and *P* value < 0.05 was considered statistically significant (24, 25).

### Weighted correlation network analysis

Co-expression networks have facilitated the development of network-based gene screening methods that can be used to identify candidate biomarkers and therapeutic targets. In this study, we constructed a gene expression data map of GSE117261 based on the WGCNA R package (26). WGCNA was used to identify genes which were related with clinical phenotype. In order to build the scale-free network, we used the function pickSoftThreshold to select soft powers β=5. An adjacency matrix was created and then transformed into a topological overlap matrix (TOM) as well as the corresponding dissimilarity (1-TOM). A hierarchical clustering tree diagram of the 1-TOM matrix was constructed to classify similar gene expressions into different gene co-expression modules. To further identify functional modules in the co-expression network, module-trait associations between modules and clinical feature information were calculated based on previous studies. As a result, modules with high correlation coefficients were considered as candidates for correlation with clinical features and were selected for subsequent analysis.

### Establishment of hypoxia-induced PH mouse model

22 ± 2 g C57BL/6 male mice were purchased from the Beijing Vital River Laboratory Animal Technology Co., Ltd. The experiment was carried out in accordance with the Guideline for Care and Use of Laboratory Animals published by the US National Institutes of Health and the Guidelines for the ethical review of laboratory animal welfare People’s Republic of China National Standard GB/T 35892-2018 (27) and approved by the Animal Ethics Committee of Shanghai Children’s Medical Center, Shanghai Jiao Tong University School of Medicine. Animals were housed at a constant temperature (21 °C) and humidity (35%) under a 12-h light/dark cycle for one-week acclimatization. The animals were then randomized into two groups. Briefly, the mice were either placed into the hypoxia chamber (Tow-int Tech, Shanghai, China) at 8.5% oxygen level or fed in ambient air with a 12-h light/dark cycle for 30 days. Sodium lime (S103651, Aladdin, Shanghai, China) was applied to absorb excess carbon dioxide to maintain a low carbon dioxide concentration of hypoxia chamber (CO_2_ ≤ 0.5%).

### Right heart catheterization

Right heart catheterization was used to evaluate the hemodynamics. Briefly, animals were subjected to isoflurane anesthesia (1.5%) by endotracheal intubation with ventilator throughout the procedure. After median thoracotomy, a fluid-filled catheter (Millar, SPR-671NR) was inserted into the right ventricle and connected to a force transducer to measure right ventricular systolic pressure.

### Magnetic resonance imaging

Cardiac MRI was performed using nuclear magnetic resonance (Bruker biospec 94/30 USR). Animals were anesthetized with 1.5% isoflurane for MRI procedure. Small animal ECG electrodes (SA Instruments) were attached to the limbs and a respiration-detection cushion was placed under the abdomen for the monitoring of ECG and respiration signals. The image acquisition was triggered by the R wave of ECG and respiration signal. After three-dimensional plane localization, two-chamber views, four-chamber views and short-axis views were captured. The MRI images were analyzed by U-Viewer software (Shanghai United Imaging Healthcare Co.,Ltd). The thickness of left and right ventricular wall were measured at the level of papillary muscle at the end of ventricular diastole phase. The analysis and calculation of MRI data was performed by two independent investigators.

### Echocardiography

The capture and measurement of echocardiography were performed using the animal’s ultrasonic machine (Vevo3100, Fujifilm). Animals were given 1.5% isoflurane and left ventricular ejection fraction was measured by M-mode at papillary muscle level under left ventricular long axis view. The level of tricuspid annular plane systolic excursion (TAPSE) was measured by M-mode under four chamber view. The measurement of echocardiography data was performed by two independent investigators.

### Hematoxylin-eosin and immunofluorescent staining

Lung tissues were fixed in 4% paraformaldehyde, paraffin-embedded and then sliced at 2 μm. For H&E staining, the paraffin-free sections were placed in an aqueous solution of hematoxylin for several minutes followed by acid and ammonia water respectively, then the sections were washed by running water and soaked with distilled water. After dehydration in 75% and 95% alcohol for 2 min each, the sections were placed into the alcohol eosin staining solution for 2-3 min. For immunofluorescent staining, the sections were dewaxed, hydrated and antigen-retrieved, and blocked with 5% donkey serum for 1 h and incubated with primary antibodies overnight at 4°C (mouse anti-ACKR4 antibody, 1:200, no. PA5106552, ThermoFisher Scientific.; mouse anti-α-smooth muscle actin, 1:300, no. 14-9760-82, eBioscience; rabbit anti-CD31, 1:300, no. ab182981, abcam). Sections were stained with fluorochrome-conjugated secondary antibodies for 60 min the next day. Images were captured under automatic fluorochrome microscope (Leica, Germany).

### Western blot

Tissue protein was extracted by RIPA buffer contained with protease inhibitors (Roche). Samples were resolved by SDS-PAGE and transferred onto nitrocellulose membranes by the iBlot 2 dry blotting system (Thermo Fisher Scientific). Transferred membranes were blocked in 5% non-fat dry milk in TBST (100 mmol/L Tris, pH 7.5, 0.9% NaCl, 0.1% Tween-20) for 1 h prior to incubation with primary antibodies against ACKR4 (mouse anti-ACKR4 antibody, 1:500, no. PA5106552, ThermoFisher Scientific) or against β-tubulin (rabbit anti-beta tubulin, 1:1000, no. ab179513, abcam) overnight at 4°C. Membranes were washed three times with TBST and incubated for 1 h with horseradish peroxidase-labeled secondary antibody at room temperature. After flushing, enhanced chemiluminescent substrate (no. WBKLS0500, Millipore) was used to develop the membranes. Image J was used to analyze the band intensities.

### Real time polymerase chain reaction

Total tissue RNA was extracted by RNA extraction kit (R701-01, Vazyme) following the manual instruction of manufacture. Reverse transcription was performed using a Primescript RT reagent kit (no. RR047Q, Takara) and RT-PCR was performed using life Technology ABI 7500 System based on SYBR-Green PCR kit (no. A25742, Thermo Fisher Scientific). The △△Ct method was used for the calculation of gene expression relative to housekeeping gene. The primer sequences for RT-PCR were listed in **Table 1**.

**Table 1:** Primers for validation by RT-PCR in mouse lungs of hypoxia induced PH.

Gene	Forward Primer	Reverse Primer
Ackr4	AACCCCATCCTGTATGTCT	CTTGTTTACTCTCTGGCGTCT
Cxcl12	CTCTCCTCAAGACAGCCGAA	CTGTGCTCCTCATCGCAAC
Col18a1	TGCCACAACAGCTACATCGTC	TTCGCCAGGAAGCTCTACCAA
Tshz2	ACACTCTGAAACAAACGACCA	TTCGACTGCCATCTCAATCCG
*Gapdh*	TCAACGACCCCTTCATTGACC	CACCAGTAGACTCCACGACA

### Statistical analysis

Bioinformatical analysis was conducted through R (version: 4.2.1) software. The least absolute shrinkage and selection operator (LASSO) method was used to select the optimal predictive features from PAH samples in GSE117261. This method is suitable for high-dimensional data reduction. Features with non-zero coefficients in the LASSO regression model were selected. Then we use the selected features to establish a linear regression model. The receiver operating characteristic (ROC) curve was used to evaluate the effectiveness of the model, and the effectiveness of the model was evaluated according to the area under the curve (AUC). All samples in GSE117261 were randomly divided into two groups, one group was used to establish the model, and the other group was used to verify the model. The samples in GSE113439 and GSE24988 were used for external verification. Statistical analysis of anatomical structure measurement, immunofluorescent staining and gene expression at mRNA or protein level were conducted with GraphPad Prism software (version 9.0.0). Differences between two groups were examined with the student *t* test. One-way ANOVA was used for 3-group comparisons. A value of *P* < 0.05 was considered statistically significant. All data were expressed as mean ± SEM.

## Results

### Identification of chemokine related DEGs in lung tissues from PAH patients

**Figure 1:**
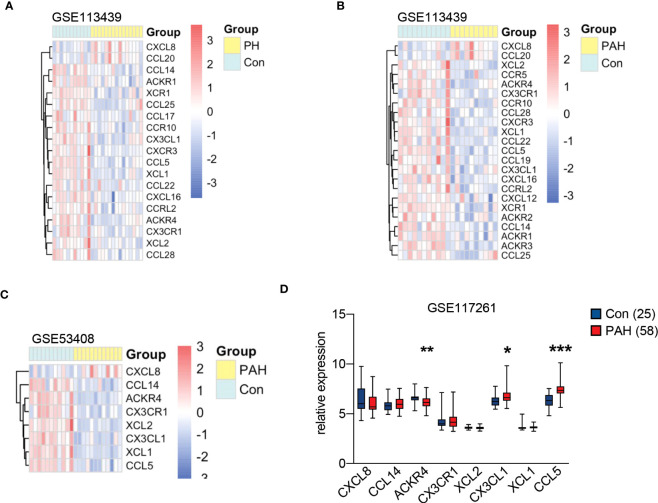
Chemokine related gene expression in pulmonary hypertension (PH) associated GEO datasets. **(A)** The heatmap of 19 chemokine related differentially expressed genes (DEGs) in lung tissues from dataset GSE113439 consisting of 11 controls and 15 PH patients. **(B)** The heatmap of 23 chemokine related DEGs in lung tissues from dataset GSE113439 consisting of 11 controls and 10 patients with pulmonary arterial hypertension (PAH). **(C)** The heatmap of 8 chemokine related DEGs in lung tissues from dataset GSE53408 consisting of 10 controls and 11 PAH patients. The red square in heatmap represents the upregulation of gene expression, and the blue square in heatmap represents the downregulation of gene expression. **(D)** The boxplot represents the gene expression of overlapped chemokine related genes in lung tissues from dataset GSE117261 consisting of 25 controls and 58 PAH. Data represent mean ± SEM. *P < 0.05; **P < 0.01; ***P < 0.001 compared to controls, as analyzed by unpaired *t* test or Mann-Whitney test as appropriate.

**Figure 2:**
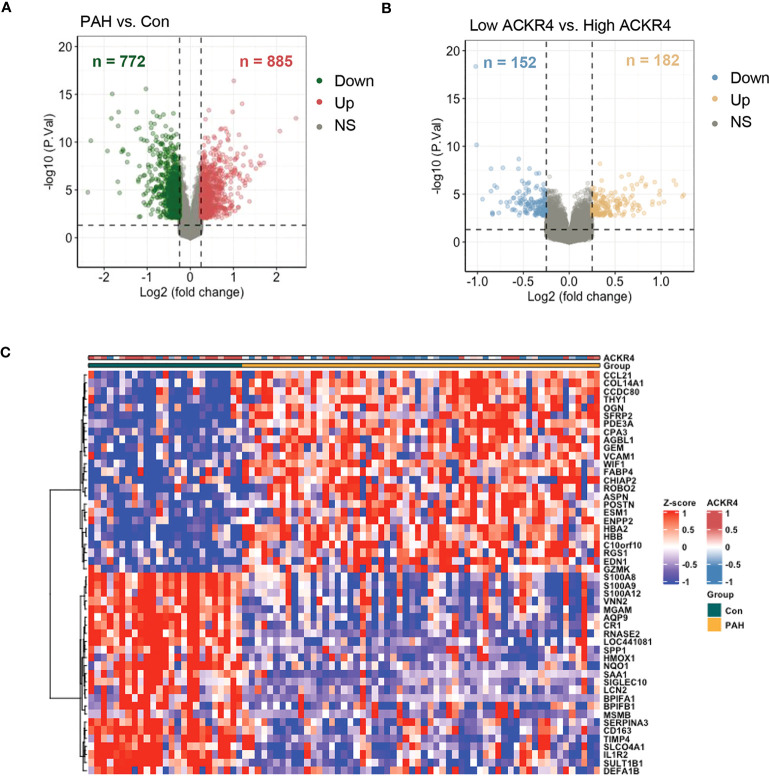
The DEG analysis in lung tissues from PAH patients in dataset GSE117261. **(A)** DEGs in lung tissues from PAH patients compared to those of controls in dataset GSE117261 were visualized in volcano plot. The red dots represent the upregulation of gene expression and green dots represent the downregulation of gene expression. **(B)** DEGs in lung tissues from PAH patients with low ACKR4 expression compared to those PAH patients with high ACKR4 in dataset GSE117261 (n=29/group; PAH patients were divided into high ACKR4 group and low ACKR4 group according to the median ACKR4 expression). The yellow dots represent the upregulation of gene expression and blue dots represent the downregulation of gene expression. **(C)** Top 25 upregulated and downregulated DEGs in lung tissues between PAH patients and controls in dataset GSE117261.

We selected chemokine related genes for differential gene analysis. A total of 19 chemokine related DEGs were screened from GSE113439 (**Figure 1A**). In GSE113439, gene expressions were examined in lung samples from 15 PAH composing of 6 idiopathic PAH, 4 PAH associated with connective tissue disease, 4 PAH associated with congenital heart disease and 1 chronic thromboembolic pulmonary hypertension. To rule out the effect caused by inflammation related connective tissue disease, we next selected the samples from 6 idiopathic PAH and 4 PAH associated with congenital heart disease to perform differential gene analysis. A total of 23 chemokine related DEGs were screened from GSE113439 subgroups (**Figure 1B**), and a total of 10 chemokine related DEGs were also screened from another PAH cohort in GSE53408 (**Figure 1C**). The intersection of the above three DEGs lists was obtained and examined in GSE117261. Finally, 3 DEGs were obtained, among which ACKR4 was downregulated, CX3CL1 and CCL5 were upregulated (**Figure 1D**). Considering that CCL5 and CX3CL1 were well studied in PAH and ACKR4 is the only gene downregulated in PAH lungs among the three genes, indicating the role of ACKR4 as a protective modulator and immune checkpoint, we therefore selected ACKR4 as the key gene for further analysis. A total of 1657 DEGs were obtained from GSE117261, which included 772 downregulated and 885 upregulated genes (**Figure 2A**). GSEA analysis showed that the lung tissue of PAH and the normal groups had completely different biological processes and pathways (**Figures S1A-B**), with PAH lung samples enriched in extracellular matrix structural constituent and innate immune system. In addition, we divided PAH samples in GSE117261 into low ACKR4 group and high ACKR4 group by median ACKR4 expression. A total of 334 DEGs were obtained, with 152 downregulated and 182 upregulated in low ACKR4 group (**Figure 2B**). Top 25 upregulated and downregulated genes in PAH patients were displayed in **Figure 2C**.

### Enrichment in low ACKR4 group and the correlation of immune infiltration with ACKR4

**Figure 3:**
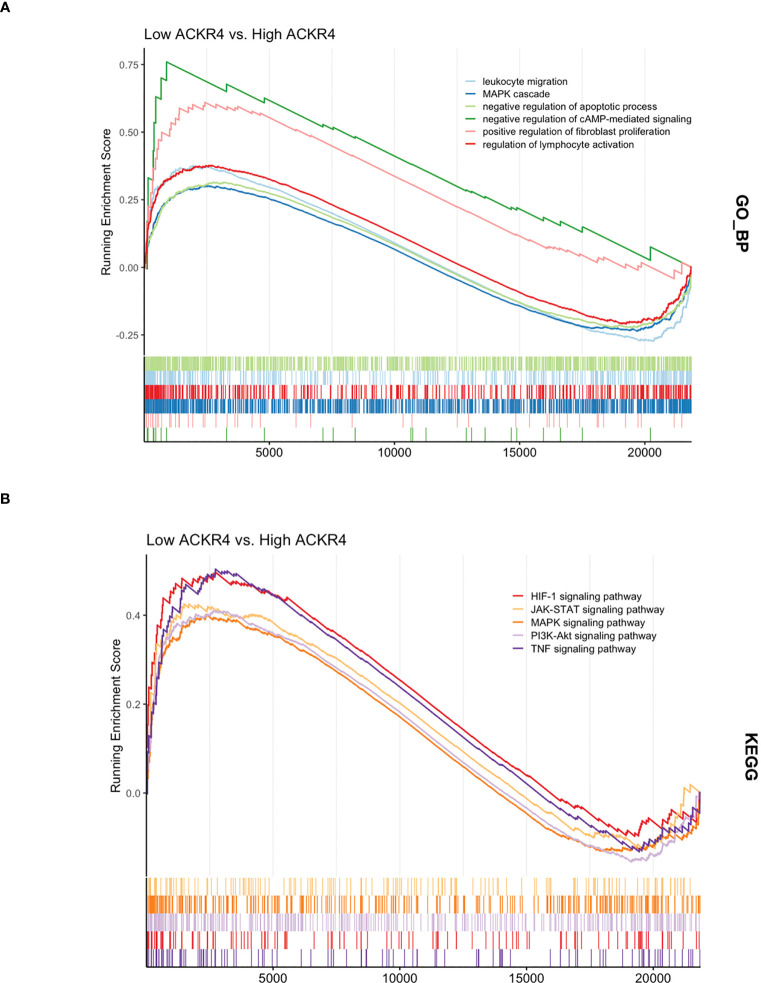
The functional and pathway enrichment in lungs from PAH patients with low ACKR4. **(A)** The Gene Ontology analysis displaying biological processes enriched in low ACKR4 group versus high ACKR4 group in dataset GSE117261. **(B)** The KEGG analysis displaying biological pathways enriched in low ACKR4 group versus high ACKR4 group in dataset GSE117261.

**Figure 4:**
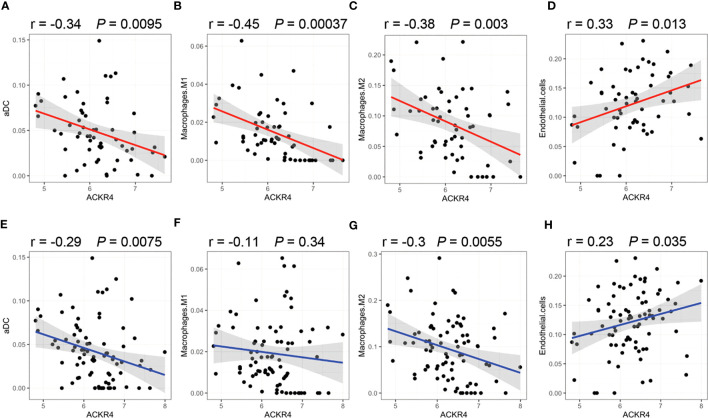
Correlation of immune infiltration with ACKR4. **(A–D)** Infiltration of activated dendritic cells (aDC), Th1 and macrophage (both M1 and M2) in lung tissues were correlated with ACKR4 expression. Data source was PAH samples from GSE117261. **(E–H)** Infiltration of aDC, Th1 and macrophage (both M1 and M2) in lung tissues were correlated with ACKR4 expression, and data source was PAH and control samples from GSE117261.

According to GSEA analysis (**Figure 3**), most genes in PAH samples with low ACKR4 were enriched in the following biological process: leukocyte migration, MAPK cascade, negative regulation of apoptotic, negative regulation of cAMP-mediated signaling, positive regulation of fibroblast proliferation and regulation of lymphocyte activation. Pathways including HIF-1 signaling pathway, JAK-STAT signaling pathway, MAPK signaling pathway, PI3K-Akt signaling pathway and TNF signaling pathway were also unraveled to be enriched in low ACKR4 PAH group. Since immune cell infiltration and endothelial cell apoptosis are hallmarks in PAH pathogenesis, we then applied xCell analysis to test the correlation between ACKR4 level and the abundance of multiple immune cells and endothelial cells. It was demonstrated that infiltration of activated dendritic cells, Th1 and macrophage (both M1 and M2) were negatively correlated with ACKR4 expression level, whereas the abundance of endothelial cells was positively correlated (**Figure 4**). Both functional or pathway enrichment and the correlation with immune cell infiltration analysis suggest a potential protective role of ACKR4 in the setting of PAH.

### Modules identified to be related to PAH *via* WGCNA analysis

**Figure 5:**
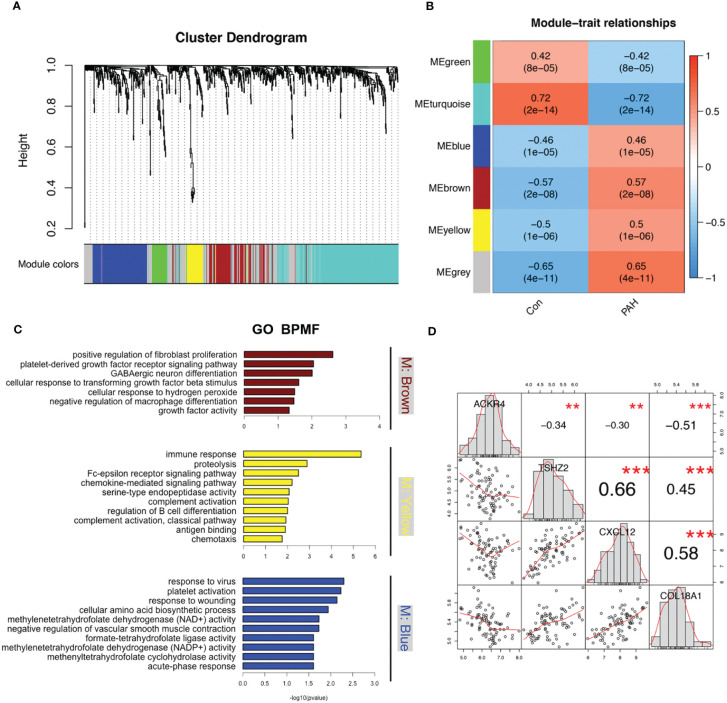
Modules related to PAH identified *via* WGCNA analysis. **(A)** The cluster dendrogram of GSE117261. Genes with the same expression characteristics will be divided into modules with the same color. **(B)** The module-trait relationships map of GSE117261. The blue module represents negative correlation with selected traits and red module represents positive correlation with selected traits. **(C)** The GO enrichments analysis of brown, yellow and blue modules. **(D)** The correlation of ACKR4, TSHZ2, CXCL12 and COL18A1.

To find disease related genes in PAH patients, we constructed a gene co-expression network using the WGCNA package. All genes were divided into different modules and each module was assigned a different color (**Figure 5A**). The correlation between each module and two clinical features was assessed and visualized *via* heatmap of module-trait relationships (**Figure 5B**), indicating that the brown, yellow and blue module in GSE117261 had high correlations with PAH [brown module (r = 0.57, *P* < 0.001), yellow module (r = 0.5, *P* < 0.001) and blue module (r = 0.46, *P* < 0.001). The results of GO demonstrated that most genes in brown module were enriched in positive regulation of fibroblast proliferation, platelet-derived growth factor receptor signaling pathway, GABAergic neuron differentiation, cellular response to transforming growth factor beta stimulus, cellular response to hydrogen peroxide, negative regulation of macrophage differentiation, growth factor activity. The most genes in yellow module were enriched in immune related function, whereas the most genes in blue module were enriched in metabolic and inflammatory response related functions (**Figure 5C**).

### Construction of diagnostic model and its validation

**Figure 6:**
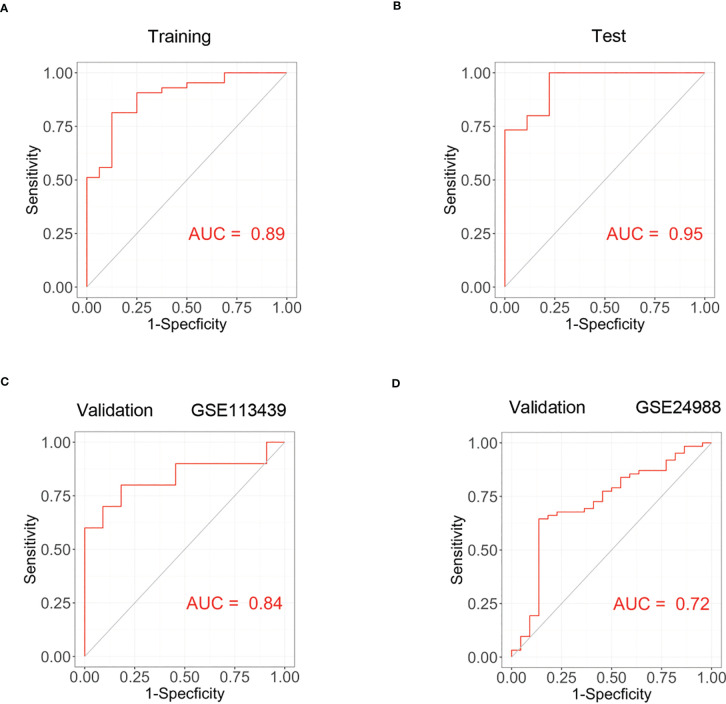
The construction of PAH diagnostic model and its validation. **(A)** The receiver operating curve of training group in dataset GSE117261. **(B)** The ROC curve of test group in dataset GSE117261. **(C)** The ROC curve of validation cohort in dataset GSE113439. **(D)** The ROC curve of validation cohort in GSE24988.

Based on genes in brown modules, 3 features including TSHZ2, CXCL12 and COL18A1 were given non-zero coefficients in the LASSO regression model. We constructed the model by following formula: Index = 0.491*COL18A1 + 0.200*TSHZ2 + 0.051*CXCL12 -3.321. We then used correlation analysis to finally determine the correlation of these three genes with ACKR4. It was demonstrated that all genes had negative correlations with ACKR4, among which COL18A1 was the most negatively correlated (**Figure 5D**). The model performed well in the training group and the validation groups. In the training group, the AUC reached 0.89 (**Figure 6A**), whereas AUC reached 0.95 in test group (**Figure 6B**). In validation cohorts, AUC reached 0.84 and 0.78 (GSE113439-**Figure 6C**, GSE24988-**Figure 6D**), respectively. This finding suggests that the diagnostic model could distinguish PAH from controls.

### Decreased ACKR4 expression in experimental PH model

**Figure 7:**
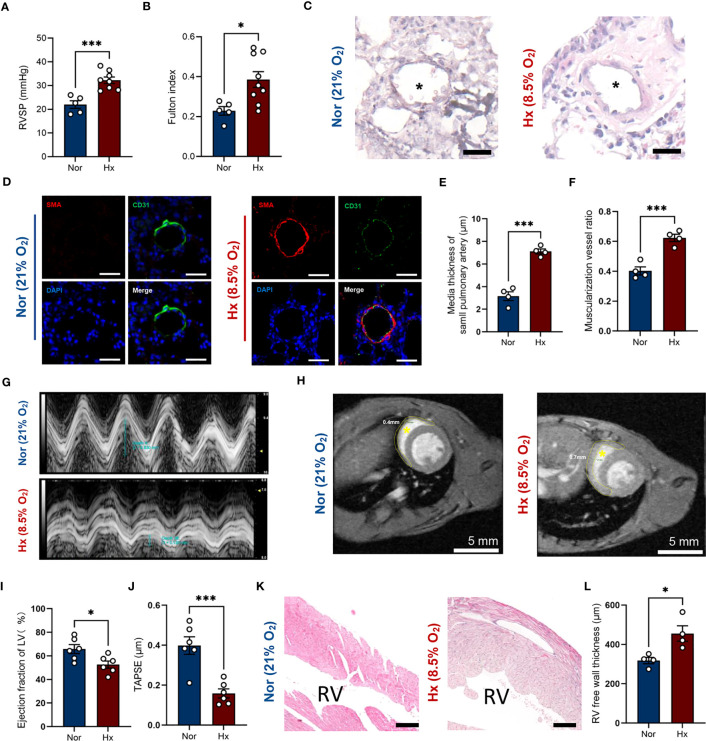
Establishment of hypoxia induced PH mouse model. **(A, B)** Right ventricular systolic pressure **(A)** and Fulton index **(B)** were assessed in hypoxia (Hx) induced PH mice or mice under normoxia (Nor) at day 30 (n=5-8/group). **(C)** Representative images of H&E staining of lung tissues in hypoxia-induced PH mice or mice under normoxia at day 30. Black asterisk indicates lumen of pulmonary artery. Scale bar = 30 μm. **(D)** Representative image of immunofluorescent staining of pulmonary arterioles. Scale bar = 30 μm. SMA = alpha smooth muscle actin. **(E, F)** Quantification of media thickness of small pulmonary arterioles **(E)** and the ratio of muscularized vessels **(F)** in lung tissues from mice under hypoxic or normoxic condition at day 30 (n=4/group). **(G)** Representative M-mode view of tricuspid annulus root. **(H)** MRI scanning of heart from mice under hypoxic or normoxic condition at day 30. Inside the yellow dashed circle is the right ventricular free wall. Yellow asterisk indicates right ventricular chamber. Scale bar = 5 mm. **(I, J)** Histograms of LV ejection fraction and TAPSE in hypoxia-induced PH mice or mice under normoxia at day 30 (n=6/group). **(K)** Representative image of H&E staining of right ventricule from mice under hypoxic or normoxic condition at day 30 (n=4/group). RV indicates right ventricular chamber. Scale bar = 150 μm. **(L)** Quantification of right ventricular free wall thickness from mice after hypoxia exposure or in ambient air (n=4/group). Data represent mean ± SEM. *P < 0.05; ***P < 0.001 compared to mice under nomorxia, as analyzed by unpaired *t* test or Mann-Whitney test as appropriate.

We established hypoxia-induced PH mouse model (8.5% oxygen, 30 days), displaying a higher right ventricular systolic pressure (32.2 ± 1.4 mmHg vs. 22.0 ± 1.6 mmHg; *P* < 0.001) and a 1.68-fold increase of Fulton index compared to that in normoxia group (**Figures 7A, B**). In addition, the media wall of pulmonary arteries in PH mice was significantly increased relative to mice in ambient air (**Figures 7C, E**). Meanwhile, the percentages of muscularized pulmonary arteries were statistically higher in hypoxia-induced PH mice (**Figures 7D, F**). The wall thickness of right ventricles in PH group was significantly thickened compared with normoxia group based on the results of MRI (**Figure 7H** and **Figures S2**, **S3**) and pathology (**Figures 7K, L**). Furthermore, we performed echocardiography to evaluate the heart function. The ejection fraction of left ventricle was slightly decreased (**Figure 7I** and **Figure S4**) and the right ventricular function was impaired evidenced by a reduction of tricuspid annular plane systolic excursion in mice after hypoxia exposure compared with normoxia group (**Figures 7G, J**).

**Figure 8:**
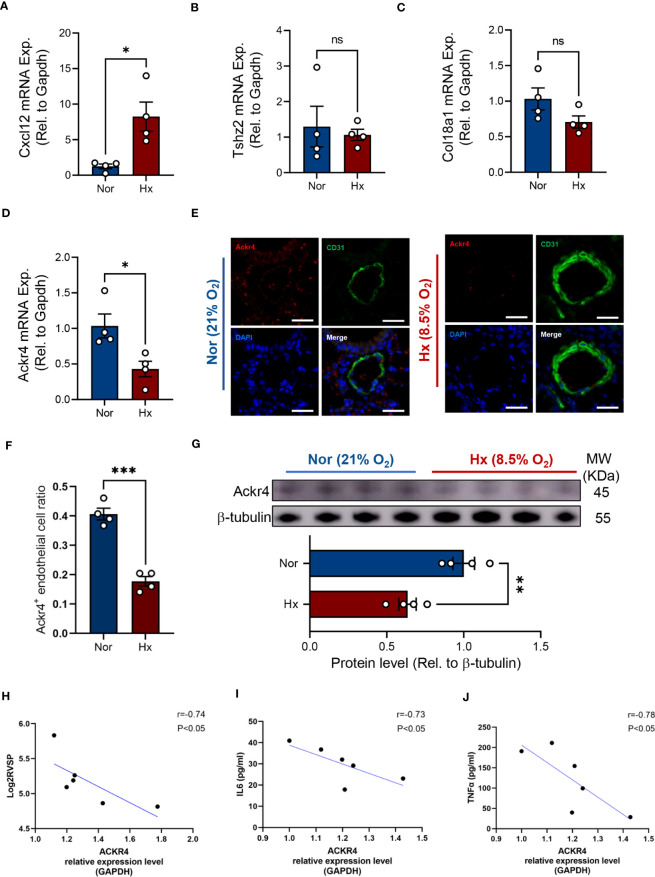
Verification of target genes in experimental PH mice. **(A–D)** The mRNA expression level of Cxcl12 **(A)**, Tshz2 **(B)**, Col18a1 **(C)** and Ackr4 **(D)** relative to housekeeping gene (Gapdh) were examined in lung tissues from hypoxia-induced PH mice or those under normoxic condition (n=4/group). **(E, F)** Representative images **(E)** of immunofluorescent staining of Ackr4 and CD31 (endothelial cell marker) and quantification of the ratio of Ackr4-expressing endothelial cells to all endothelial cells **(F)** in lung tissues from hypoxia-induced PH mice or those under normoxic condition (n=4/group). Scale bar = 30 μm. **(G)** Representative immunoblotting bands of Ackr4 in lung tissues and its difference between mice after hypoxia and those under normoxia (n=4/group). Ackr4 expression at protein level was normalized to β-tubulin. Data represent mean ± SEM. *P < 0.05; **P < 0.01; ***P < 0.001 compared to mice under nomorxia, as analyzed by unpaired *t* test or Mann-Whitney test as appropriate. **(H–J)** Correlation analysis of ACKR4 mRNA expression in lung tissues with log2- transformed right ventricular systolic pressure (Log2RVSP) **(H)**, interleukin-6 (IL6) **(I)**, and tissue necrosis factor (TNF)-α **(J)**. ns, not significant.

Next, we examined the mRNA expression of the three genes determined by WGCNA analysis in lung tissues of PH mice. It turned out that *Cxcl12* was significantly increased in lungs after hypoxia exposure (**Figures 8A–C**). Of note, *Ackr4* mRNA expression decreased almost 2-fold in lung tissues of PH mice (**Figure 8D**). In consistent with previous finding, fewer Ackr4^+^ endothelial cells were documented in lung tissues of PH mice as well compared with normoxia mice (**Figures 8E, F**). Similarly, the protein level of Ackr4 expression in lungs of PH mice was about two thirds of that in normoxia mice (**Figure 8G**). Additionally, ACKR4 expression at RNA level in lung tissues had strong negative correlations with log2-transformed right ventricular systolic pressure, as well as interleukin 6 (IL-6) and tissue necrosis factor (TNF)-α levels in lung homogenates (*P* < 0.05, All correlation coefficient > 0.7) (**Figures 8H–J**). This would suggest that ACKR4 might modulate the severity and inflammation of PAH.

## Discussion

Inflammation plays a central role in the development of PAH, and the recruitment and function of immune cells are tightly orchestrated *via* various chemokines. However, the chemokine network in lungs from multiple PAH cohorts remains largely undefined, especially those responsible for the inflammation resolvement. In this study, we first determined that atypical chemokine receptor 4 encoded by ACKR4 was downregulated in PAH lung tissues in all selected datasets by differential gene analysis. Subsequently, PAH relevant biological functions and pathways were enriched in patients with low-ACKR4 level according to GSEA enrichment analysis. The negative correlation of multiple immune cell infiltration with ACKR4 expression was also revealed. We screened three gene modules associated with PAH using WGCNA analysis, and finally constructed a diagnostic model to distinguish PAH from control subjects. The expression level of ACKR4 was also downregulated in lung tissues of our experimental PH mice compared to that of controls. Of note, ACKR4 expression had strong correlations with log2-transformed right ventricular systolic pressure as well as the inflammatory cytokines (IL6 and TNF-α) in lung homogenates. Our findings provide evidence in support of the notion that ACKR4 might serve as a previously unrecognized protective immune checkpoint of the pathological vascular remodeling in PAH.

The protein encoded by ACKR4 is a member of the G protein-coupled receptor family and is a receptor for C-C type chemokines (28). This receptor has been shown to bind chemokines activated by dendritic cells and T cells, including CCL19/ELC, CCL21/SLC and CCL25/TECK. Interestingly, ACKR4 is an atypical chemokine receptor that controls chemokine levels by binding with high affinity to chemokines, which leads to reduced levels of chemokines and resolved inflammation (29–31). It plays an important role in the migration of immune and tumor cells. Some studies have linked ACKR4 to cancer and immune deficiency disease (11, 32). In this study, it is proposed for the first time that ACKR4 may be related to PAH and might be a new target for the treatment of the disease.

CXCL12 encodes a protein that is inducible to lymphocytes and monocytes and acts as a positive regulator of monocyte migration (33). CXCL12 is a ligand that binds to CXCR4 as well as ACKR3, thus allowing monocytes to migrate in a specific direction, causing a local inflammatory response (34, 35). In this study, it was confirmed that CXCL12 expression levels were significantly upregulated in lung tissues from PH mice, indicating a close relationship between CXCL12 and PAH. This is in line with previous studies pertaining to the detrimental role of CXCL12 in pulmonary vascular remodeling and the fact that neutralization of CXCL12 attenuates experimental PH (36, 37). Our previous study in combination of metabolomics and transcriptomics of human pulmonary artery smooth muscle cells also showed that CXCR4, one of recognized receptors for CXCL12, was identified as metabolism associated gene responded to proliferating stimuli and positively correlated with immune cell infiltration (18). The blockade of CXCL12-CXCR4 axis was shown to mitigate the progression of PAH in β-catenin dependent manner (38). In contrast, ACKR4 expression level was downregulated in PAH group, which may indicate that down-regulation of ACKR4 expression levels resulted in the limitation of control of chemokine levels. Dysregulated CCL21 and CCL19, ligands of ACKR4, were also revealed in human PAH and CCL21 appears to be a potential biomarker for predicting the risk of PAH in systemic sclerosis (39, 40). As CXCL12 is predominantly expressed by lung endothelial cells and induced in PAH (41, 42). The negative correlation of ACKR4 with CXCL12 might indicate that downregulation of ACKR4 in endothelial cells might affect angiogenesis, cell survival or other functions, thus exacerbating PAH development. However, how the reduction of ACKR4 leads to the elevation of CXCL12 remains unclear and vice versa, which warrants further investigation in future studies to decipher a more comprehensive chemokine-related atlas in the setting of PAH.

We eventually developed a model for the diagnosis of PAH that incorporated TSHZ2, CXCL12 and COL18A1 as predictors. The predictive ability of this model performed well in the training group as well as in validation cohort using another two datasets. This model can guide clinicians in the diagnosis of PAH. However, this model has shortcomings. Experimental validation of the three predictors in the model by RT-PCR revealed that only the expression level of CXCL12 was statistically significant in both groups. This may be related to the different environmental adaptations among species. Mice are more capable of environmental adaptation, and the experimental use of a hypoxic chamber to construct a PH model in mice may lead to insignificant differences in the expression levels of TSHZ2 and COL18A1.

Some limitations should also be noted in this study. First, it is difficult to obtain human PAH lungs for the verification of ACKR4 alteration. Second, we had no access to more detailed clinical characteristics such as hemodynamics in the datasets. Additionally, we were unable to discern the causality between ACKR4 and immune cell abundance due to the cross-sectional design of the datasets. However, we used multiple datasets of PAH lungs to examine chemokine related genes and the reduction of ACKR4 was consistent among all the involved datasets, which was also in line with the ACKR4 alteration in PH mouse model. The role of ACKR4 in the progression of PAH warrants further investigation.

In conclusion, our results suggest that ACKR4 might be instrumental in PAH, targeting ACKR4 may represent a promising strategy in the intervention of PAH.

## Data availability statement

The datasets presented in this study can be found in online repositories. The names of the repository/repositories and accession number(s) can be found below: https://www.ncbi.nlm.nih.gov/geo/, GSE117261; https://www.ncbi.nlm.nih.gov/geo/, GSE113439; https://www.ncbi.nlm.nih.gov/geo/, GSE53408; https://www.ncbi.nlm.nih.gov/geo/, GSE24988.

## Ethics statement

The animal study was reviewed and approved by Animal Ethics Committee of Shanghai Children’s Medical Center, Shanghai Jiao Tong University School of Medicine (Shanghai, China).

## Author contributions

C-YJ and L-WW carried out animal experiment, contributed to data acquisition, analysis and drafted the manuscript. Y-WL, BF, and L-CY contributed to data interpretation and manuscript revision. XH and Y-YH performed right heart catheterization for mice phenotyping. YS performed RT-PCR for gene validation. Y-FZ, X-LZ, and D-JJ contributed to data interpretation and provided crucial intellectual support. H-KQ contributed to MRI data acquisition and supervised the study. HZ and YY conceived and supervised the study and revised the manuscript. All authors contributed to the article and approved the submitted version.
