# Corrigendum: Identification of ACKR4 as an immune checkpoint in pulmonary arterial hypertension

**DOI:** 10.3389/fimmu.2024.1382832

**Published:** 2024-02-20

**Authors:** Chen-Yu Jiang, Li-Wei Wu, Yi-Wei Liu, Bei Feng, Lin-Cai Ye, Xu Huang, Yang-Yang He, Yi Shen, Yi-Fan Zhu, Xing-Liang Zhou, Dai-Ji Jiang, Hai-Kun Qi, Hao Zhang, Yi Yan

**Affiliations:** ^1^ Shanghai Clinical Research Center for Rare Pediatric Diseases, Shanghai Children’s Medical Center (SCMC), School of Medicine, Shanghai Jiao Tong University, Shanghai, China; ^2^ Heart Center and Shanghai Institute of Pediatric Congenital Heart Disease, Shanghai Children’s Medical Center (SCMC), School of Medicine, Shanghai Jiao Tong University, Shanghai, China; ^3^ School of Pharmacy, Henan University, Kaifeng, Henan, China; ^4^ School of Biomedical Engineering, Shanghaitech University, Shanghai, China

**Keywords:** pulmonary arterial hypertension, atypical chemokine receptor 4, transcriptomics, chemokines, immune

In the original article, there was a mistake in the **Funding** statement as published. The Projects of National Natural Science Foundation of China (82200065, 82000064) were wrong. The correct **Funding** statement appears below.

FUNDING

“This work was supported by the Project of National Natural Science Foundation of China (U20A2018), the Project of Shanghai Municipal Science and Technology Commission (20MC1920400), the National Key Research and Development Program of China (2022YFC2703102), Projects of National Natural Science Foundation of China (82200065, 82241007), Shanghai Pujiang Program (22PJ1410100), Young Talent Program of Shanghai Municipal Health Commission (2022YQ070) and Innovative research team of high-level local universities in Shanghai.”

The authors apologize for this error and state that this does not change the scientific conclusions of the article in any way.

