# Supplementary material for: Identification of ACKR4 as an immune checkpoint in pulmonary arterial hypertension
Source: Front Immunol. 2023 Jun 28;14:1153573. doi: 10.3389/fimmu.2023.1153573 (PMC10337759; doi:10.3389/fimmu.2023.1153573)

Supplementary Materials

Identification of ACKR4 as an immune checkpoint in pulmonary arterial hypertension

Supplementary Table 1. Chemokine related genes examined in this study
Supplementary Figure 1. Gene set enrichment analysis of GO and REACTOME in lung tissues from PAH versus controls in dataset of GSE117261
Supplementary Figure 2. Full MRI sequence images of normoxia mouse heart
Supplementary Figure 3. Full MRI sequence images of hypoxia mouse heart
Supplementary Figure 4. Representative M-mode view of left ventricular chamber

Supplementary Table 1. Chemokine related genes examined in this study.

Genes encoding ligands			Genes encoding receptors	
CCL1	CCL19	CXCL3	CCR1	CXCR5
CCL2	CCL20	PF4	CCR2	CXCR6
CCL3	CCL21	CXCL5	CCR3	ACKR1
CCL4	CCL22	CXCL6	CCR4	ACKR2
CCL5	CCL23	CXCL8	CCR5	ACKR3
CCL7	CCL24	CXCL9	CCR6	ACKR4
CCL8	CCL25	CXCL10	CCR7	CCRL2
CCL11	CCL27	CXCL11	CCR8	XCR1
CCL13	CCL28	CXCL12	CCR9	CX3CR1
CCL14	CCL3L1	CXCL13	CCR10	
CCL15	CCL3L3	CXCL14	CXCR1	
CCL16	CX3CL1	CXCL16	CXCR2	
CCL17	CXCL1	XCL1	CXCR3	
CCL18	CXCL2	XCL2	CXCR4	

Supplementary Figure 1. Gene set enrichment analysis of GO and REACTOME in lung tissues from PAH versus controls in dataset of GSE117261.

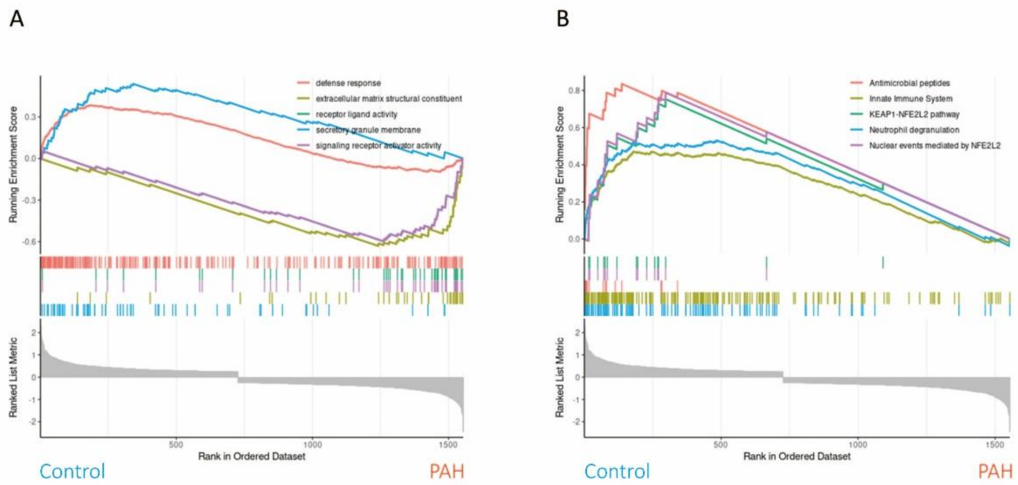

Supplementary Figure 2. Full MRI sequence images of normoxia mouse heart

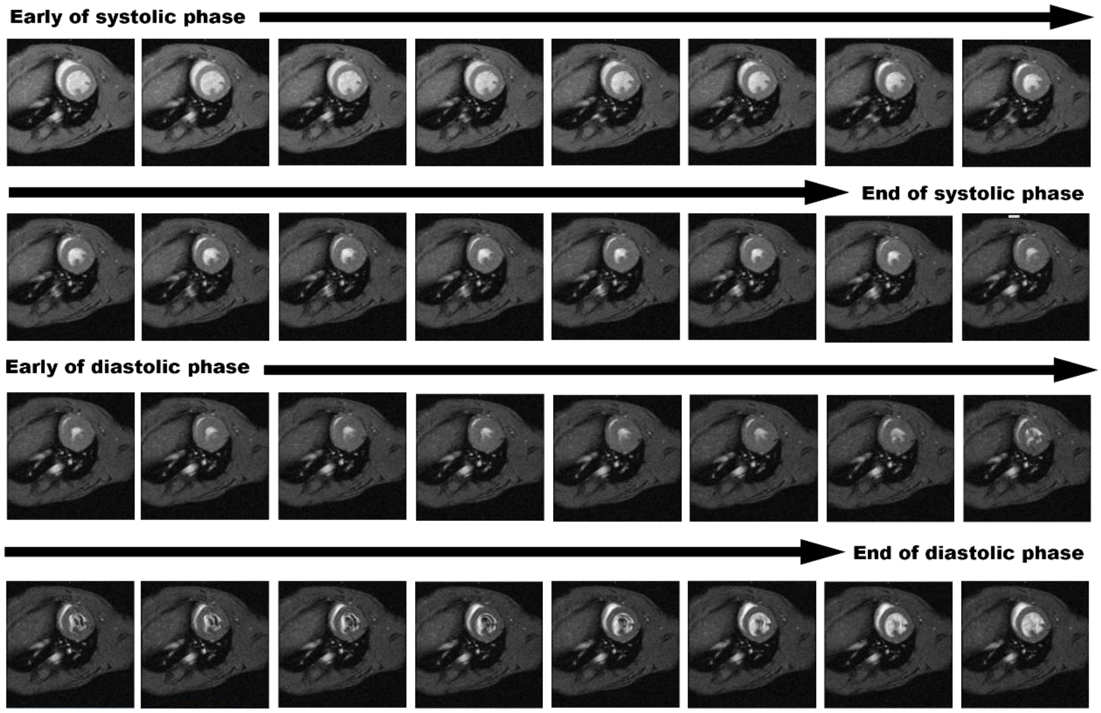

Supplementary Figure 3. Full MRI sequence images of hypoxia mice heart

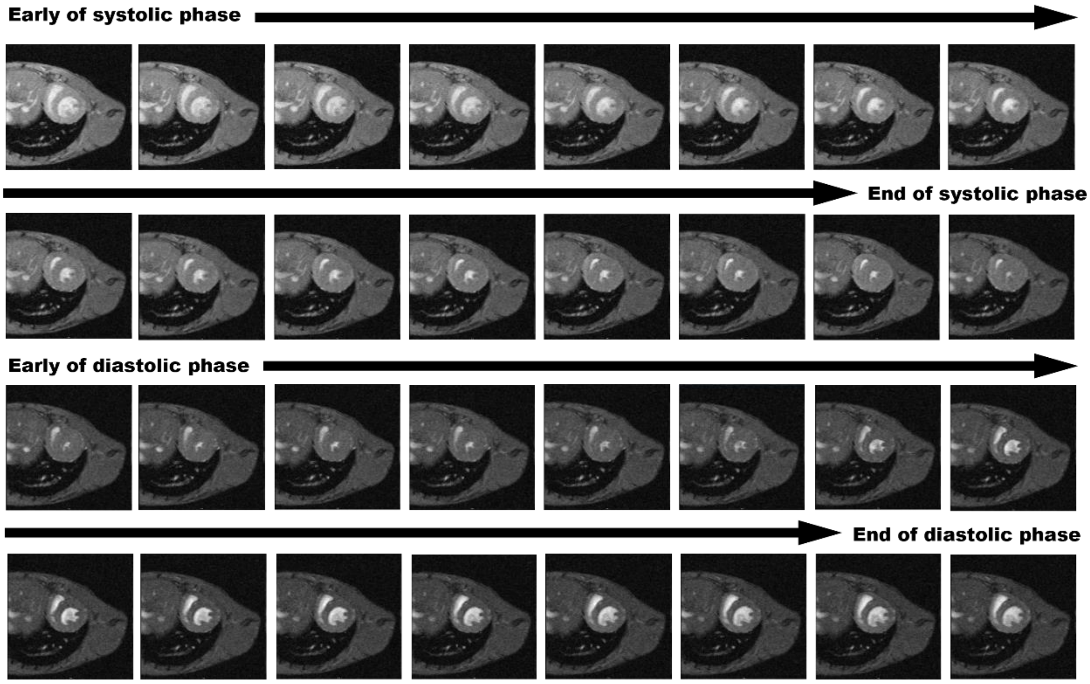

Supplementary Figure 4. Representative M-mode view of left ventricular chamber.

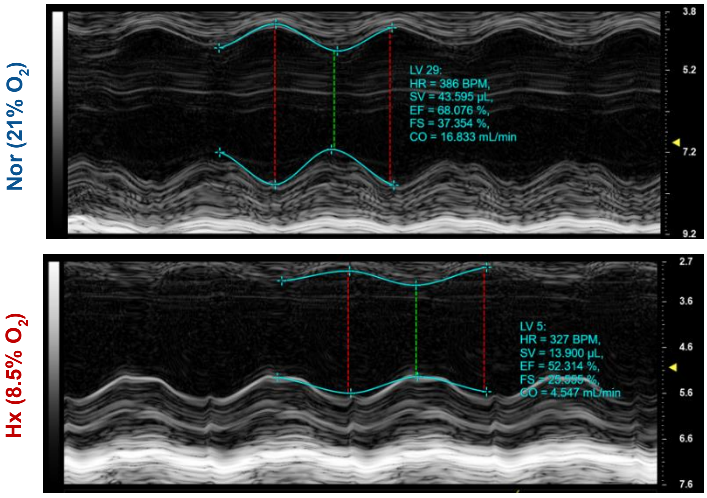

Supplement: Supplementary file 1 [file DataSheet_1.pdf]
